# Update on the Treatment of Metastatic Urothelial Carcinoma

**DOI:** 10.1155/2018/5682078

**Published:** 2018-06-06

**Authors:** Nedal Bukhari, Humaid O. Al-Shamsi, Faisal Azam

**Affiliations:** ^1^Department of Medical Oncology, King Fahad Specialist Hospital Dammam, Dammam, Saudi Arabia; ^2^Associate Professor, University of Sharjah, Sharjah, UAE

## Abstract

Platinum-based combination chemotherapy has been the standard of care in the first-line treatment of metastatic urothelial carcinoma (mUC). Treatment of metastatic disease following progression on platinum-based regimens has evolved significantly in the last few years. Clinical trials are currently ongoing to determine how best to use and sequence these treatments. In this minireview, we will review current first-line treatment options in both cisplatin fit and cisplatin unfit patients and advances in first- and second-line treatments including chemotherapy and immunotherapy. This review reports key findings from the clinical trials especially highlighting the importance of PD-1 and PD-L1 inhibitors in the treatment of bladder/urothelial carcinomas.

## 1. Introduction

Bladder cancer is the ninth most common cancer worldwide, with 430 000 diagnosed in 2012. More than 60% of all bladder cancer cases and half of all the 165000 bladder cancer deaths occur in the less developed regions of the world. A strong male predominance is observed with almost 75% of all bladder cancer cases occurring in men [1].

Major risk factors for bladder cancer include older age, male gender, Caucasian race, personal/family history of bladder cancer, smoking, and exposure to aromatic amines, such as benzidine and beta-naphthylamine used in the dye industry, dietary supplements containing aristolochic acid, and arsenic in drinking water [2]. Prior cyclophosphamide chemotherapy and radiation therapy are well-recognised risk factors [1, 2]. Mutations in the retinoblastoma gene, phosphatase and tensin homolog (PTEN) gene and Lynch syndrome are also associated with bladder cancer [2].

Approximately 25% of patients with a bladder cancer have muscle-invasive disease. These patients will either present or develop subsequently metastatic disease [3]. Systemic chemotherapy is the standard initial treatment for patients with inoperable locally advanced mUC. Initial response rates are usually high and the median survival with chemotherapy is approximately 15 months. For patients with advanced unresectable or metastatic disease, treatment with a platinum-based regimen has been the cornerstone of treatment. However novel agents have been introduced recently and are showing promising results [4].

## 2. First-Line Treatment

### 2.1. Chemotherapy

First-line treatment with cisplatin-based chemotherapy specifically cisplatin-gemcitabine (GC) regimen and the combination of methotrexate, vinblastine, doxorubicin, and cisplatin (MVAC) regimen have been the standard of care for many years [4]. MVAC was introduced as first-line chemotherapy in the early 1990s based on results from studies by Logothetis et al. and Loehrer et al. Toxicity and toxicity-related hospital admission are a major concern with MVAC therapy, mainly, myelosuppression, profound neutropenia, sepsis, mucositis, nausea, and vomiting [5–7].The use of hematopoietic growth factor support can reduce the risk of developing these toxicities, especially myelosuppression and mucositis [5–7].

GC has become the most popular first-line chemotherapy regimen in Europe and North America based on Von der Maase trial results, which demonstrated similar survival between patients treated with GC versus MVAC with better safety profile [8], although response rates to these regimens are high but not durable with median overall survival of only 12 to 15 months for patients with advanced disease[3, 8].

The addition of paclitaxel to cisplatin and gemcitabine (PCG) in metastatic and unresectable locally advanced bladder/urothelial carcinoma resulted in a higher response rate and a 3.1-month survival benefit compared to GC. These results were statistically not significant [9].

Presence of liver metastasis, hemoglobin level of less than 10 g/dL, and performance status of more than zero are adverse prognostic factors identified by Bellmunt et al. and had a significant impact on OS. Four subgroups were formed based on the presence of zero, one, two, or three prognostic factors; the median OS times for these subgroups were 14.2, 7.3, 3.8, and 1.7 months, respectively [12].

A significant proportion of mUC cancer patients is not fit for cisplatin chemotherapy. Cisplatin unfit patients were defined based on results of a survey of genitourinary medical oncologists [13]. According to this definition, unfit patients would meet at least one of the following criteria:

- Eastern Cooperative Oncology Group (ECOG) performance status ≥ 2
- Creatinine clearance < 60 mL/min
- Grade ≥ 2 hearing loss
- Grade ≥ 2 neuropathy

Reversible causes of renal insufficiency, like urinary tract obstruction, should be excluded and treated before rendering patient cisplatin unfit [13].

For patients who are not fit for cisplatin, treatment options include both chemotherapy and immunotherapy. Carboplatin and gemcitabine have shown to be a reasonable alternative to cisplatin in metastatic UC. It is noteworthy that carboplatin has an inferior response rate compared to cisplatin however has the advantage of being easily administered and better tolerated. The benefit of carboplatin-based therapy was demonstrated in EORTC trial 30986 [10, 14, 15]. Regimens combining gemcitabine with a taxane (either paclitaxel or docetaxel) rather than platinum have been evaluated with some antitumor activity [15]. An Italian multicenter trial looked at gemcitabine and paclitaxel combination in 54 cisplatin unfit patients showed an ORR of 37% in an intent-to-treat analysis.

The median survival was 13.2 months, and the median time to disease progression was 5.8 months. Single-agent chemotherapy has not demonstrated any survival advantage in clinical trials [16–21].

### 2.2. Immunotherapy

Immunotherapy is changing the treatment landscape of many cancers. Immune check point inhibitors especially antiPD-1 and antiPD-L1 monoclonal antibodies are the most effective immunotherapeutic agents for the treatment of UC. These agents showed significant antitumor, tolerable safety profiles, and durable, long-term responses in subset of patients with mUC.

*2.2.1*. Pembrolizumab is a PD-1 check point inhibitor approved in first line for patients unfit for cisplatin. In a single arm phase II study (Keynote-52) pembrolizumab had shown an objective response rate (ORR) of 24% with 7% complete responses and 22% partial responses and 18% had stable disease as best response. Median time to response was only 2 months (range: 1-9) and 82% of responses lasted more than 6 months. The ORR was higher in patients with PD-L1 expression >10 %, but responses were also observed in those with PD-L1 expression <10 %. median OS at 6 months was 67% [22].

*2.2.2*. Atezolizumab is another immune checkpoint inhibitor approved for first-line treatment of patients with mUC and not fit for cisplatin. Unlike pembrolizumab this acts as a PD-L1 inhibitor. It was tested in phase II study (IMVIGOR 210). It had 2 cohorts with cohort 1 tested Atezolizumab in first-line setting and cohort 2 tested in second-line setting. ORR for cohort 1 of this study reported an ORR of 23.5% with 5 % complete response after a median follow-up of 8.5 months. Grade 3-4 adverse events were reported in 12% and all grade treatment-related adverse events were reported in 64%. Discontinuation due to treatment-related adverse events was reported in 4.2% patients [23].

## 3. Second-Line Treatment

### 3.1. Immunotherapy

Immune check point inhibitors using PD-1 inhibitors and PD-L1 inhibitors are now the standard of care for the treatment of mUC in second-line setting after cisplatin-based chemotherapy.

*3.1.1*. Pembrolizumab gained FDA approval after results of Keynote 045, a phase III trial, where 542 patients who were previously treated with a platinum-based chemotherapy got randomized to pembrolizumab or investigator's choice of chemotherapy using paclitaxel, docetaxel, or vinflunine. The primary endpoints were OS and PFS in the entire population and those with a combined positive score (CPS) 10% for PD-L1 expression (Table 2). In the OS analysis of patients with combined positive score 10%, there was a 43% reduction in the risk of death with pembrolizumab compared with standard chemotherapy (HR, 0.57; 95% CI, 0.37–0.88; p= 0.0048). The median OS was 8.0 months (95% CI, 5.0–12.3 months) with pembrolizumab versus 5.2 months (95% CI, 4.0–7.4 months) with chemotherapy. Longer duration of response (DOR) was seen with pembrolizumab compared to that with chemotherapy; median DOR was not reached, and 68% of responders were considered likely to maintain a response for 12 months. On the contrary, the median DOR in the chemotherapy arm was 4.3 months with an estimated 35% likely to maintain a response for 12 months. Pembrolizumab was better tolerated with fewer adverse events (AEs) as compared to chemotherapy. This trial was the first to report the OS benefit with immunotherapy agent over an active comparator in advanced/metastatic UC. A number of trials are currently investigating this agent in combination with other agents for UC [24].

*3.1.2*. Atezolizumab's efficacy has been proven in 2 clinical trials. Its approval was based on a single arm, multinational phase II study (IMvigor 210) with 316 patients, which showed significant ORR and durability of responses. Patients in cohort 2 of this study were those whose tumors were progressing after first-line platinum-based chemotherapy. The primary outcome of ORR was obtained in 15% (45/310) of patients with 5% obtaining a complete response (CR). Median OS was 7.9 months (95% CI 6.7-9.3). For patients with high PD-L1 expression on infiltrating immune cells (IC2/3), a median OS and 12 months of OS were 11.9 months and 50%, respectively, as compared to 6.7 months and 31% in patients with IC 0/1. Atezolizumab was very well tolerated, grade 3–4 TRAEs, of which fatigue was the most common, occurring in 16% of treated patients. Grade 3–4 immune-mediated adverse events occurred in 5% of treated patients. There were no treatment-related deaths [25, 26].

Another clinical trial, a phase III (IMVIGOR 211) trial randomized 234 patients to Atezolizumab or investigators choice of chemotherapy. This study failed to meet the primary endpoint of improving OS in patients with high PD-L1 expression. Median OS was 11.1 months with Atezolizumab as compared to 10.6 months with chemotherapy (HR 0.87, 95% CI 0.63-1.2, and p=0.41) [23, 24], although primary endpoint of the study was not met but median duration of response in intention to treat (ITT) group was higher with Atezolizumab as compared to chemotherapy (21.7% vs. 7.4%). Responses were also more durable with Atezolizumab as compared to chemotherapy (63% vs. 21%) at the time of data cut-off. It was also better tolerated than chemotherapy with Grade 3-4 AE reported in 43% of patients in chemotherapy arm and 20% patients in Atezolizumab arm [25, 26].

*3.1.3*. Nivolumab is a fully human IgG4 monoclonal antibody against PD-1 and is approved for patients with advanced UC who were previously treated with a platinum-based regimen. It has been studied in subsequent line setting in the phase I/II open-label, multicenter CheckMate 032 study. Primary endpoint was ORR and secondary endpoints were safety, DOR, PFS, and OS. A confirmed ORR was 24.4%. Grade 3–4 treatment-related side effects (TRAEs) occurred in 22% patients. Serious AEs occurred in 46% patients of which 10% were treatment related. 3% of patients discontinued treatment and died due to treatment-related grade 4 pneumonitis and thrombocytopenia.

Checkmate 275 was a larger single arm phase 2 study. It recruited 275 patients with mUC who progressed on a platinum-based regimen. Nivolumab showed an ORR of 19.6% and responses were seen at all levels of PD-L1 expression. The median time to response was 1.9 months. Median OS was 8.74 months overall (5.95 months in patients with PD-L1 <1% and 11.3 months in patients with PD-L1 expression of 1%). Grade 3–4 TRAEs occurred in 18% of patients. Fatigue and diarrhea were most common. Quality of life improved from baseline and remained stable through the trial. Nivolumab in combination with other immunotherapy agent, including ipilimumab, is currently being studied in several clinical trials [27, 28].

*3.1.4*. Durvalumab is another IgG1 monoclonal antibody targeting PD-L1. It was approved after phase I/II dose escalation and dose expansion study reported its safety and efficacy in patients with metastatic urothelial carcinoma who progressed during or after platinum-based chemotherapy.

The primary endpoint was safety and secondary endpoints were ORR and disease control rate at 12 weeks. Sixty-one patients were enrolled, 40 of whom were PD-L1 positive. 63.9% experienced TRAEs. A majority of TRAEs were low grade and grade 3 TRAEs occurred in three patients (4.9%). Most common were diarrhea, pruritus, and infusion-related reactions. The ORR was 31%; in the PD-L1 positive subgroup and 0% in the PD-L1 negative subgroup. The disease control rate, which includes those with ORR and stable disease at 12 weeks, was 57.1% in the PD-L1 positive subgroup and 28.6% in the PD-L1 negative subgroup. Based on these promising results, durvalumab was granted breakthrough therapy designation for patients with PD-L1 positive patients with inoperable or mUC that had previously progressed on a standard platinum-based regimen. Durvalumab is being studied in several trials in UC, alone or in combination with tremelimumab [29].

*3.1.5*. Avelumab is a fully human IgG1 monoclonal antibody against PD-L1. It is approved for the subsequent line treatment of advanced UC. Approval was based on phase 1b JAVELIN trial that included patients pretreated with a platinum-based regimen. A recent ORR was reported at 17.3% irrespective of PD-L1 expression. Fatigue, weakness, infusion-related reactions, and nausea were the most common side effects [27]. Phase III trial of avelumab as maintenance therapy after first-line platinum-based therapy for advanced urothelial carcinoma is ongoing [30, 31].

### 3.2. Chemotherapy

Although a significant number of patients have an OR to first-line therapy, most eventually progress. Second-line chemotherapy may be indicated for those who are not candidates for immunotherapy and for those who progress during or after immunotherapy. There are multiple chemotherapeutic agents that have antitumor activity after progressing on either MVAC or GC as demonstrated in clinical trials that were mostly phase II. Those agents include vinflunine, which is approved in Europe, paclitaxel, docetaxel, nab-paclitaxel, gemcitabine, and pemetrexed [32–34].

*Combination Approaches and Targeted Treatments*. Molecular analysis and subtyping have identified numerous genetic and epigenetic alterations in UCs; examples include mutations in some receptor tyrosine kinases (RTK*s*) RAS/RAF, PI3K, AKT, and mammalian target of rapamycin (mTOR) pathways. Other mutations also identified are TP53, RB1, FGFR3, CCND1, MDM2, PTEN deletions, FGFR 1 amplifications, and aberrations of the chromatin remodeling genes [35].

Several clinical trials have looked at and some are currently investigating targeting these mutations. Although targeted treatments do not have an established role, single-patient benefit has been reported [35].

An ongoing phase 2 clinical trial, NCI-MATCH (ClinicalTrials.gov Identifier NCT02465060), is currently evaluating 19 likely actionable somatic mutations in advanced refractory solid tumors, lymphomas, or multiple myeloma [36].

A recent phase 1 study looked at delivering enfortumab vedotin, an antibody-drug conjugate targeting Nectin-4, a protein expressed in UC. This drug demonstrated a favourable tolerability profile with encouraging antitumor activity in heavily pretreated mUC patients [37].

Although the checkpoint inhibitors are active, not all patients will respond. Strategies to enhance the immune response by combining immunotherapy with immune sensitizers such as chemotherapy, immunotherapy, or radiation are being actively explored [38].

A phase III Keynote-36 is an ongoing trial looking at pembrolizumab ± chemotherapy versus chemotherapy in first-line setting. Patients will be randomly assigned 1:1:1 to pembrolizumab, pembrolizumab + chemotherapy (either Cisplatin-Gemcitabine OR Carboplatin-Gemcitabine if cisplatin ineligible), or chemotherapy alone. The primary endpoints are PFS and OS. Secondary endpoints are ORR and safety [38].

Angiogenesis inhibition has also emerged as an attractive strategy in UC; a combination of chemotherapy and anti-VEGF, docetaxel, and ramucirumab was associated with a better PFS and ORR when compared to docetaxel in mUC patients pretreated with a platinum-based regimen; this was based on early results from a phase III trial [39].

Combining anti-CTLA-4 and anti-PD-L1 is also being investigated. DANUBE is a phase III trial looking at durvalumab monotherapy vs. durvalumab combination therapy with tremelimumab vs. chemotherapy (Cis/Gem or Carbo/Gem) in stage IV urothelial carcinoma (Table 1). Whether combination strategies are safe and more effective than single agents remains to be seen [40].

## 4. Discussion

Chemotherapy has been the mainstay of first- and second-line treatment in mUC. Recent advances have made the newer therapies for patients with mUC. Novel immunotherapy agents have shown significant antitumor activity, tolerable safety profiles, and durable, long-term responses in clinical trials. Atezolizumab, avelumab, durvalumab, nivolumab, and pembrolizumab are promising PD-1/PD-L1 blockade drugs that are changing the standard of care in mUC. Biomarkers, prognostic factors, and molecular subtyping might play a major role in predicting responses to these drugs. Recent whole genome mRNA expression profiling studies revealed that bladder cancers can be grouped into molecular subtypes, mainly basal and luminal, with each enriched with specific alterations and copy number aberrations that are thought to underlie their distinct progression patterns, biological and clinical behavior [39]. Several groups have used different strategies to this aim, with partially overlapping findings. The results confirmed that alterations involving RB1 and NFE2L2 were enriched in basal cancers, whereas alterations involving FGFR3 and KDM6A were enriched in luminal tumors [41].

Five new immunotherapy treatments have been recently approved. These new agents approved in mUC were monoclonal antibodies against PD-1 including nivolumab and pembrolizumab or monoclonal antibodies blocking PD-L1 including Atezolizumab, durvalumab and avelumab [11].

These agents have been generally well tolerated with a side effect profile quite different than that of chemotherapy. Grade 3–4 AEs have been similar in the 15–18% range in each of the five agents. Immune-mediated adverse events have been lower, in approximately 2–5% range, and include pneumonitis, transaminitis, rash, thyroid abnormalities, and colitis [41]. In the first-line setting, 8% of patients had to discontinue Atezolizumab due to treatment-related toxicity. One notable difference is that 20.5% of patients receiving avelumab had a grade 1-2 infusion-related reaction [42, 43]. The quality of life for patients improved on nivolumab and remained stable with treatment. In phase III randomized pembrolizumab data, rate of adverse events was significantly lower in the pembrolizumab group compared to the chemotherapy group (60.9 vs. 90.2%) [24].

Much work is ongoing to understand molecular characteristics to predict benefit with chemotherapy and immunotherapy in UC. Treatment with Atezolizumab resulted in more responses in luminal cluster 2 tumors, while treatment with nivolumab suggested that basal type 1 tumors (Cluster III) had the most benefit. However, both datasets suggested low levels of activity in luminal 1 tumors. Luminal 1 (Cluster II) tumors appear to lack the immune markers expressed in luminal 2 and at even higher levels in basal tumors. The enrichment for FGFR3 mutations in the luminal 1 subtype suggests the potential for FGFR3 inhibitors as a future treatment in these immunologically cold tumors. Clinical activity also appeared lower in the basal 2 tumors, a group with the highest levels of expression of immune markers. OR with immune checkpoint inhibitors remains in approximately 15–20% range, suggesting many still do not benefit from these therapies. Although some might consider transitioning from a PD-L1 to a PD-1 inhibitor or vice versa upon progression, it seems unlikely that the immune stimulatory effects observed with these single agents will overcome resistance observed with prior PD-1/PD-L1 blockade. Pairing CTLA-4 blockade along with PD-1/PD-L1 blockade has been effective in other tumor types to enhance the immune response and is being further explored in UCs in multiple ongoing clinical trials [42].

Members of the tumor necrosis factor receptor (TNFR) family such as OX40 and 4-1BB may serve as potential targets for novel treatments. Targeting OX40 and 4-1BB with agonist antibodies elicits potent antitumor responses; however further investigating these agents is required [44, 45].

## 5. Conclusion

Cisplatin-based combination remains the standard in the first-line treatment of mUC. Immunotherapy and chemotherapy are active options in first line for cisplatin unfit patients. Immunotherapy emerged as promising treatment in second-line setting.

It is clear that we are just at the beginning of the new immune era in the treatment of cancer. The field is progressing with hope to answer existing questions, including optimal place in treatment, duration of use, predictive biomarkers, patient selection, and cost. The only way to answer these questions is to continue to accrue patients for clinical trials, which has led us to where we are today.

## Figures and Tables

**Table 1:** Treatment options for metastatic urothelial carcinoma [10].

First-line cisplatin eligible	Cis/Gem, MVAC.

First-line cisplatin ineligible	Chemotherapy: Carbo/Gem, single agent chemotherapy.
	Immunotherapy: Atezolizumab or Pembrolizumab.
	Clinical trial

Second line	Immunotherapy: Pembrolizumab, Atezolizumab, Nivolumab, Durvalumab and Avelumab.
	Chemotherapy: single agent Vinflunine, Taxanes (Paclitaxel, nab-paclitaxel and Docetaxel), Gemcitabine, Pemetrexed.
	Clinical trial

**Table 2:** Immunotherapy trials [11].

Setting	Drug	Study	No. of patients	ORR %	ORR (%) by PD-L1 Subgroup (high/low)	PD-L1 High +ve Prevalence (%)
Post platinum	Atezolizumab	IMvigor210	310	15	26 (IC 2/3) 32	32
					9 (IC 0/1)	
	Nivolumab	Checkmate 275	270	19.6	28.4 (TC ≥ 5%)	30
					16.1 (TC*<* 1%)	
	Pembrolizumab	KEYNOTE 045	542	21	21.6 (CPS ≥ 10%)	30
					NR	
	Durvalumab	Study 1108	103	20	31 (TC or IC ≥ 25%)	59
					5 (TC and IC*<* 25%)	
	Avelumab	JAVELIN	44	18	53.8 (TC ≥ 5%)	35
					4.2 (TC *<* 5%)	

Cisplatin ineligible	Atezolizumab	IMvigor210	119	23	28 (IC 2/3)	27
					21 (IC 0/1)	
	Pembrolizumab	KEYNOTE 052	370	24	39 (CPS ≥ 10%)^*∗*^	35

*Abbreviations*. CPS: combined positive score (assesses PD-L1 expression as a composite of both IC and TC expression); IC: immune cell; NR: not reported; ORR: objective response rate by Response Evaluation Criteria in Solid Tumors (RECIST) version 1.1; PD-L1: programmed death receptor ligand-1; TC: tumor cell. ^*∗*^CPS ≥ 10% as an expression cutoff was defined in the first 100 patients; the ORR of 39% is reported for the validation set of the remaining 270 patients enrolled.
